# Noise Source Visualization for Small DC Motors Using Current Reference without a Reference Microphone

**DOI:** 10.3390/mi9060290

**Published:** 2018-06-09

**Authors:** Yong Thung Cho

**Affiliations:** Division of Mechanical and Automotive Engineering, Kongju National University, ChunAn, ChoongNam 31080, Korea; cho.yong@gmail.com; Tel.: +82-41-521-9277

**Keywords:** motor noise visualization, micro motor, motor current, moving reference

## Abstract

Noise and vibration sources from small direct current (DC) motors should be clearly visualized for optimal design of low noise motors. For accurate visualization, relatively good reference measurements at optimal locations are required. For some very small motors, the optimal position for a stationary reference microphone may not be accessible during measurement. However, strategies for small motor noise visualization without using a reference microphone have been developed in this study. Only scanning microphones and current measurements of a small motor were used to visualize sound sources. Scanning microphone signals combined with current measurements were used as moving reference signals. Motor noise visualization results based on different moving reference locations have been estimated and reported. Consistent motor noise visualization results from motor current and different, moving reference locations for the major electro-magnetic force excitation frequencies have been shown. Furthermore, for frequencies with relatively low current amplitude, clear motor noise visualization results have been produced for a moving reference located at the center of the motor. Also, the relationship between motor noise and current has been shown, and motor noise has been reduced by connecting an optimal capacitor to the motor power input.

## 1. Introduction

Various analytical and experimental efforts to design and manufacture low noise motors have been made. The electro-magnetic force and cogging torque of these motors has been actively analyzed [1,2,3], and noise radiating from structural and acoustical resonances was measured and matched through simulation [4]. The relationship between motor current and radiated noise was investigated both in simulation and by experiment [5]. To derive an optimal design for low noise motors, sources of motor noise and vibration should be accurately visualized. Such sources were clearly visualized for a small direct current (DC) motor, and the results were confirmed with structural excitation tests using an impact hammer and motor run-up tests for different motor rotational speeds [6]. For accurate visualization of motor noise sources, relatively good reference measurements at optimal locations close to the sources is required [7,8]. In previous work, a reference microphone was located above the motor shaft to avoid physical interference with scanning microphones [6]. However, as motors are constructed to be increasingly smaller, the optimal position for a stationary reference microphone may not be accessible for actual measurement. In this research, strategies for small motor noise visualization without using a reference microphone have been explored. Due to the possibility of spatial interference between scanning and reference microphones as size of motors and distance between source and measurement surfaces were very small, it would be ideal to visualize very small noise sources with only scanning microphone measurements (eliminating reference microphone measurements). Only scanning microphones and current measurements for a motor were used to visualize sound sources. First, the current measurement of the motor was used as a reference signal for noise visualization, with scanning microphone measurements. However, the amplitude of the current measurement decreased very rapidly as frequency increased, so scanning microphone signals were combined with current measurements and used as moving reference signals. Motor noise visualization results based on different moving reference locations were estimated and presented in this study.

For major electro-magnetic force excitation frequencies, the consistency of motor noise visualization results from motor current and different moving reference locations were investigated. Also, improvements in motor noise visualization results were investigated for frequencies with relatively low current amplitude, using moving references located in various positions around the motor. Moreover, the possibility of motor noise reduction with an optimal capacitor connected to the motor power input by reducing the commutator arc was quantified. Additionally, the relationship between motor current and sound pressure at different locations for run-up operation of the motor at different rotational speeds was investigated.

In this study, noise sources for a small DC motor were investigated based on sound pressure measurement using scanning microphones and motor current. The measurement of motor current was used as a reference signal and combined with sound pressure measurements to estimate moving reference signals at various locations around the motor to improve sound visualization results. This method for sound visualization was determined to be appropriate for improving sound visualization for very small motors when reference microphones cannot be placed at optimal locations close to sources.

## 2. Partial Fields and Transfer Functions between Sources and Sound Measurement

For sound visualization of a source to be accurate, measurement of the sound field should be coherent. Otherwise, measurement pressure should be decomposed into coherent partial fields [7]. Partial field decomposition should also be used if the total measurement surface should be scanned with relatively few microphones and appropriate reference signals. The formulation for partial field decomposition starts by relating the source, reference, and field signals. Reference signals, **R**, and field microphones signals, **Y**, can be expressed using transfer functions to represent their relationship with source signals [7,8,9]:

**R** = **G**_rs_**s** (1)

**Y** = **G**_ys_**s** (2)

where **s** represents source signals, and **G**_rs_ and **G**_ys_ are transfer functions between a source and reference, and a source and field signals, respectively. The cross-spectral matrix between reference signals and field microphone signals, **S**_ry_, and the spectral matrices of the reference and field microphones signals, **S**_rr_ and **S**_yy_, can be written as

**S**_ry_ = E{**RY**^H^} = **G**_rs_**S**_ss_**G**_ys_^H^ (3)

**S**_rr_ = E{**RR**^H^} = **G**_rs_**S**_ss_**G**_rs_^H^ (4)

**S**_yy_ = E{**YY**^H^} = **G**_ys_**S**_ss_**G**_ys_^H^ (5)

The Hermitian of the cross-spectral matrix and the inverse of the reference spectral matrix are

**S**_ry_^H^ = **G**_ys_**S**_ss_**G**_rs_^H^ (6)

**S**_rr_^−1^ = (**G**_rs_^H^)^−1^**S**_ss_^−1^**G**_rs_^−1^ (7)

In addition, **G**_ys_ can be calculated by post-multiplying **S**_ry_^H^ by (**G**_rs_^H^)^−1^**S**_ss_^−1^, i.e.,

**G**_ys_ = **S**_ry_^H^(**G**_rs_^H^)^−1^**S**_ss_^−1^ (8)

and the transfer function between the reference and field microphones, **H**_yr_, is then

**H**_yr_ = **YR^−^**^1^ = **G**_ys_**G**_rs_**^−^**^1^ = **S**_ry_^H^(**G**_rs_^H^)^−1^**S**_ss_^−1^**G**_rs_^−1^ = **S**_ry_^H^**S**_rr_^−1^ (9)

Thus, the transfer function, **H**_yr_, depends on the source and reference geometry. It is independent of source level. The autospectral matrix of the field microphones, **S**_yy_, is then

**S**_yy_ = **G**_ys_**S**_ss_**G**_ys_^H^ = **H**_yr_**G**_rs_**S**_ss_**G**_ys_^H^ = **H**_yr_**S**_ry_ = **S**_ry_^H^**S**_rr_^−1^**S**_ry_ = **PP**^H^ (10)

Hence, the partial field matrix, **P**, is

**P** = **S**_ry_^H^**S**_rr_^−1/2^ = **H**_yr_**S**_rr_^1/2^ = **G**_ys_**S**_ss_^1/2^ (11)

The partial field matrix is considered to represent measurement pressure for noise source visualization in this research. The moving reference signal, **R**_m_, is defined such that

**R**_m_ = **G**_rms_**s** (12)

where **G**_rms_ is the transfer function between the source and a moving reference. The moving reference is located closer to the source than a typical reference location. The transfer function, **H**_yr_, can now be represented in terms of the moving reference as

**H**_yr_ = **YR^−^**^1^ = **Y R**_m_**^−^**^1^**R**_m_**R^−^**^1^ = **G**_ys_**G**_rms_**^−^**^1^**G**_rms_**G**_rs_**^−^**^1^ = **H**_yr__m_**H**_r__mr_ (13)

where **H**_yrm_ is the transfer function between a moving reference and field microphones. Similarly, **H**_rmr_ is the transfer function between a stationary reference and moving reference. The partial field matrix, **P**, based on a moving reference is

**P** = **H**_yr_**S**_rr_^1/2^ = **H**_yr__m_**H**_r__mr_**S**_rr_^1/2^ = **S**_r__my_^H^**S**_r__mr__m_^−1^**S**_rr__m_^H^**S**_rr_^−1^**S**_rr_^1/2^ = **S**_r__my_^H^**S**_r__mr__m_^−1^**S**_rr__m_^H^**S**_rr_^−1^^/2^ = **G**_ys_**S**_ss_^1/2^ (14)

In this context, sound pressure measurement taken via scanning microphones was used as the moving reference signal and motor current measurement was used as the fixed reference.

## 3. Sound Visualization

An acoustical holography procedure [10], which also could be described as an inverse system procedure, was implemented to visualize motor noise with current measurement as a reference. Acoustical holography was first introduced for projecting measurements with spherical coordinates, followed by a version with cylindrical coordinates [11,12]. An alternative holography procedure, statistically optimized near-field acoustical holography (SONAH), was derived to reduce spatial truncation and the size of the measurement surface [13,14]. SONAH was also modified for the projection of measurements with cylindrical coordinates [15]. Cylindrical SONAH was implemented to visualize noise radiated from power seat slide motors and small DC motors [16]. By using fixed reference signals during measurement, a scan could be completed with a relatively small number of microphones for successful acoustical holography reconstruction [7,17]. In this study, cylindrical SONAH was implemented to identify noise sources in a small DC motor with motor current used as a reference signal and sound pressure measurements used as both scanning and moving reference signals. A more detailed derivation of cylindrical SONAH has been provided in a previous work [6,15], so SONAH is described relatively briefly here.

Cylindrical SONAH is based on the assumption that sound pressure *p*(**r**) at an arbitrary position can be represented as a linear combination of measured sound pressure data, $p(r_{h,j})$ [15]:

(15) $$p(r)\approx \sum_{j=1}^{J}c_{j}(r)\cdot p(r_{h,j})$$

Sound pressure on a cylindrical surface of radius *r* can be expressed as

(16) $$p(r,\phi ,z)=\sum_{m=-\infty }^{m=\infty }\frac{1}{2\pi }\int_{-\infty }^{\infty }P_{m}(r,k_{z})e^{im\phi }e^{ik_{z}z}dk_{z}$$

where *P_m_*(*r*,*k_z_*) is the cylindrical wave number spectrum of *p_m_*(*r*,*φ*,*z*) for the *m*th circumferential component of the sound field, and *k_z_* is the axial component of the wave number. The wave number spectrum at radius *r* can also be expressed in terms of the wave number spectrum of the sound field on a cylindrical source surface of radius *r_s_*:

(17) $$P_{m}(r,k_{z})=\frac{H_{m}^{\left(1\right)}(k_{r}r)}{H_{m}^{\left(1\right)}(k_{r}r_{s})}P_{m}(r_{s},k_{z})$$

where $H_{m}^{\left(1\right)}$ is the *m*th order Hankel function and the radial wave number is

(18) $$k_{r}=[\begin{array}{l} \sqrt{k^{2}-k_{z}{}^{2}}\;\mathrm{for}\;\left|k\right|\geq \left|k_{z}\right|\\ i\sqrt{k_{z}{}^{2}-k^{2}}\;\mathrm{for}\;\left|k\right|<\left|k_{z}\right| \end{array}$$

The three-dimensional cylindrical wave function,$\Phi_{k_{z},m}(r,\phi ,z)$, was defined as

(19) $$\Phi_{k_{z},m}(r)=\Phi_{k_{z},m}(r,\phi ,z)\equiv \frac{H_{m}^{\left(1\right)}(k_{r}r)}{H_{m}^{\left(1\right)}(k_{r}r_{s})}e^{im\phi }e^{ik_{z}z},\;r\geq r_{s}$$

The same coefficients *c_j_* in Equation (15) also provide a good estimation for the cylindrical wave functions:

(20) $$\Phi_{k_{zq},m}(r)\approx \sum_{j=1}^{J}c_{j}(r)\Phi_{k_{zq},m}(r_{h,j}),\;m=1\ldots M,\;q=1\ldots N$$

Coefficients *c_j_* can be estimated from the cylindrical wave functions at measurement and reconstruction locations by using statistically optimal or least square solutions. Radial particle velocity on the reconstruction surface, *u_r_*(**r**), can be found using Euler’s equation:

(21) $$u_{r}(r)=\frac{1}{i\rho_{o}\omega }\frac{\partial p(r)}{\partial r}$$

Radial particle velocity, *u_r_*(**r**), is estimated from the spatial distribution of sound pressure as

(22) $$u_{r}(r)\approx p^{T}(r_{h})d(r)$$

where **d**(**r**) is the transfer matrix between measurement pressure and reconstructed particle velocity.

Pressure and particle velocity can be reconstructed at other surfaces, such as source surfaces, from measurement pressure. More detailed and accurate information about the source can be obtained using reconstructed properties than with measurement pressure alone.

## 4. Measurement Description

**Figure 1:**
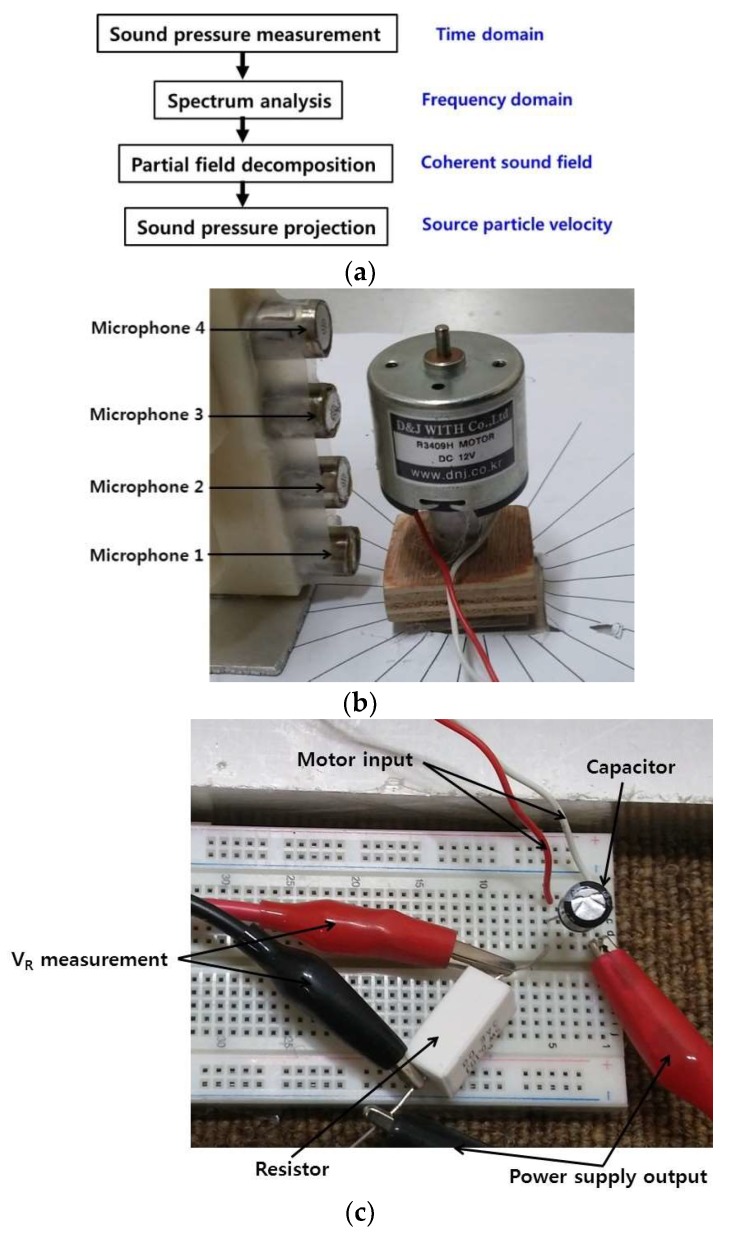
Overall motor noise measurement and visualization: (**a**) measurement and visualization procedure (**b**) motor and microphones; (**c**) capacitor and resistor.

**Figure 2:**
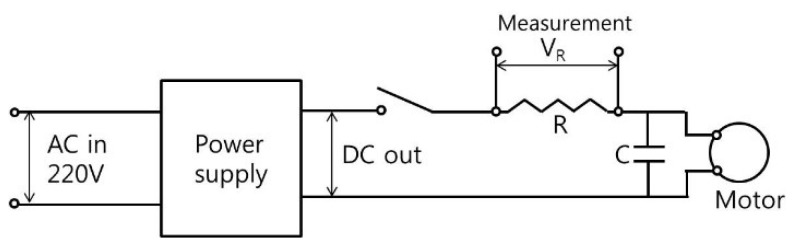
Motor power supply and resistor for measurement of current provided to the motor.

Sound pressure and current for a motor rotating at constant speed were measured on a cylindrical surface to enable reconstruction of the particle velocity of a source surface. The measurement of sound pressure was achieved in a way similar to in previous work, where it is described in detail [6]. The same motor and scanning microphones were used for measurement, but reference signals were taken from motor current. For measurements taken in previous studies, reference signals were taken from a reference microphone located above the motor [6]. However, there was no reference microphone used in this work, and motor current was measured to provide reference signals instead. This approach for taking measurements without a reference microphone is useful for visualization of noise radiated from very small motors and other micro structures. For example, if the size of the motor is smaller than that of a microphone, there will be no space for a reference microphone to be placed. Furthermore, one critical reason for using the same motor as in previous research was so sound visualization results could be compared and confirmed more conveniently when using different reference signals for the same motor. Overall schematic of the system for measurement and sound source visualization is shown in Figure 1a. The motor and microphones used for noise measurement are shown in Figure 1b. Microphones 1 to 4 were located sequentially from the bottom to top of the motor, as shown in Figure 1b, which represents zero degrees and has each scan rotated 15 degrees counter-clockwise (CCW). The number of measurements taken to cover the complete circumference was twenty four. The spacing between the microphones was 2 cm, and the distance from each microphone to the surface of the motor was 1 cm. The resistor and capacitor connected between the power supply and motor input are shown in Figure 1c. The voltage of the resistor shown in Figure 1c was measured to estimate the motor current. Also, a diagram showing how the motor power supply was connected to a resistor for measuring the current provided to the motor is shown in Figure 2.

In addition to scanning the entire surface of the motor housing for noise visualization, sound pressure was also measured in a run-up test with microphones located at zero degrees. Power supply from 6 V to 15 V in increments of 0.3 V was provided to the motor for the run-up tests. Similarly, sound pressure was measured with various capacitors at the zero-degree microphone location.

A total of four scanning microphones were used for the measurements taken in this study. Electret condenser microphones powered with a 9 V battery were used as scanning microphones and more details of the microphones were described in previous work [17]. The microphones were calibrated at 1000 Hz, 94 dB, and connected to a microphone amplifier. Output from the microphone amplifier and voltage measurements from the resistor to measure motor current were directed to an analog digital converter which was connected to a laptop computer, and the measurement data was saved on the laptop. All signals were sampled at 44.1 kHz. The Hann window was applied to all microphone and current measurements, except for the results shown in the time domain. For discrete Fourier transform, frame size of 11,025 points, overlap of 50%, and frequency resolution of 4 Hz was applied for all results.

## 5. Measurement Results

Motor noise and current were measured under motor run-up conditions with different rotational speeds by varying the motor input voltage, which was useful for characterizing the noise directly related to rotational forces and structural or acoustical resonances. Also, motor noise and current were measured under a constant motor rotational speed of 4960 rpm at 12 V, with various different capacitors as shown in Figure 1 and Figure 2 to reduce motor noise related to brush and commutator arcs by installing various capacitances in the motor power input. Finally, sound pressure and current for a motor rotating at constant speed were measured on a cylindrical surface, scanning the entire surface of the motor to reconstruct the particle velocity of source surfaces. Measurements were taken for a motor rotating both counter-clockwise (CCW) and clockwise (CW) at a constant speed of 4960 rpm and 5080 rpm, respectively. There was slight deviation in motor rotational speed when the motor was rotating in different directions, but the nominal power supply output voltage remained the same, 12 V. The values for resistance and capacitance for measuring current and reducing arcs in the motor, as shown in Figure 1 and Figure 2, were 0.1 Ω and 100 μF for all measurements presented in this research unless described otherwise.

**Figure 3:**
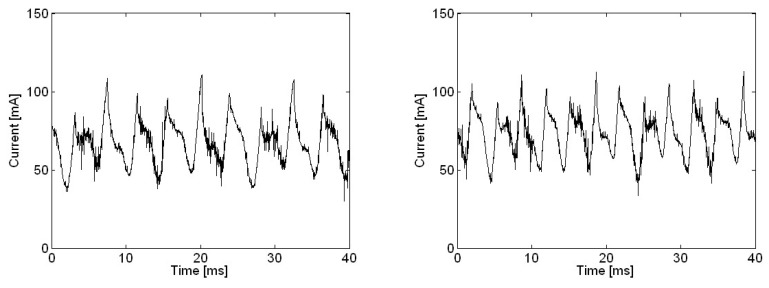

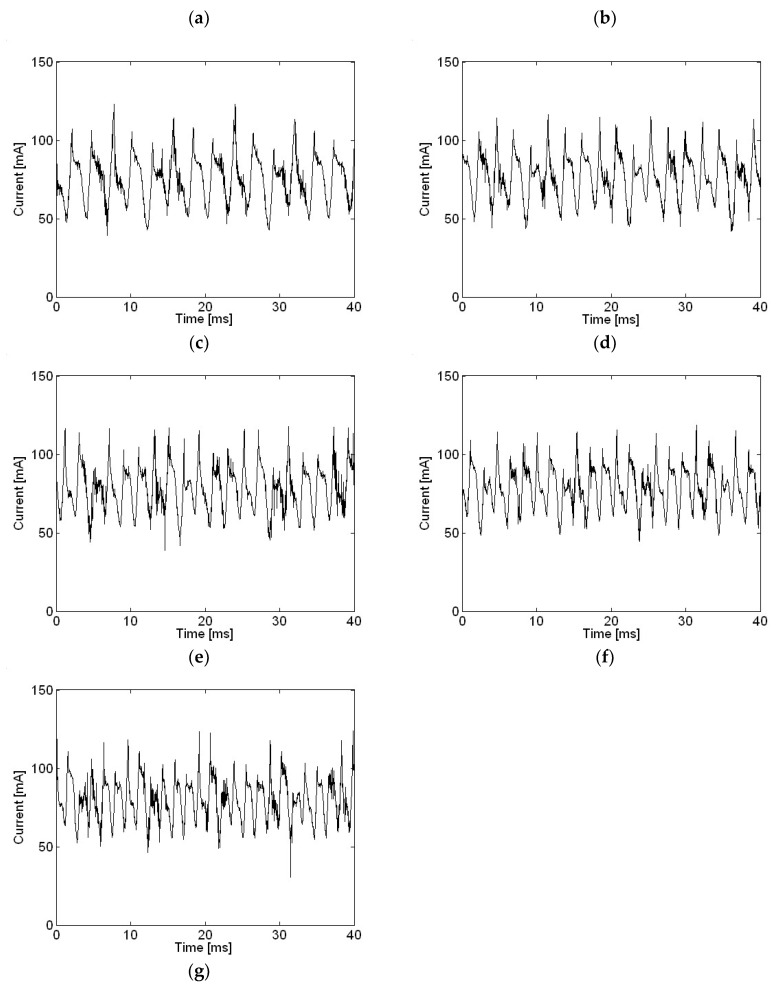
Motor current measurement in the time domain with various motor rotational speeds: (**a**) 2480 rpm; (**b**) 3100 rpm; (**c**) 3720 rpm; (**d**) 4340 rpm; (**e**) 4960 rpm; (**f**) 5580 rpm; (**g**) 6200 rpm.

Motor current measurements in the time domain with various motor rotational speeds were taken by adjusting power supply voltage as shown in Figure 3. There were two brushes and three commutators poles, such that every sixth waveform corresponded to the same brush and commutator pole; every third waveform corresponded to the same commutator pole; and every second waveform corresponded to the same brush. Even for the low motor speed of 2480 rpm, the current measurement waveform deviated significantly from the harmonic waveform. One major reason for this deviation could be the relatively small size of the commutator, which was 6.25 mm in diameter, and a lack of precision manufacturing. For even smaller commutators, as in smaller motors, the waveform for motor current would be likely to deviate even more from the harmonic waveform.

**Figure 4:**
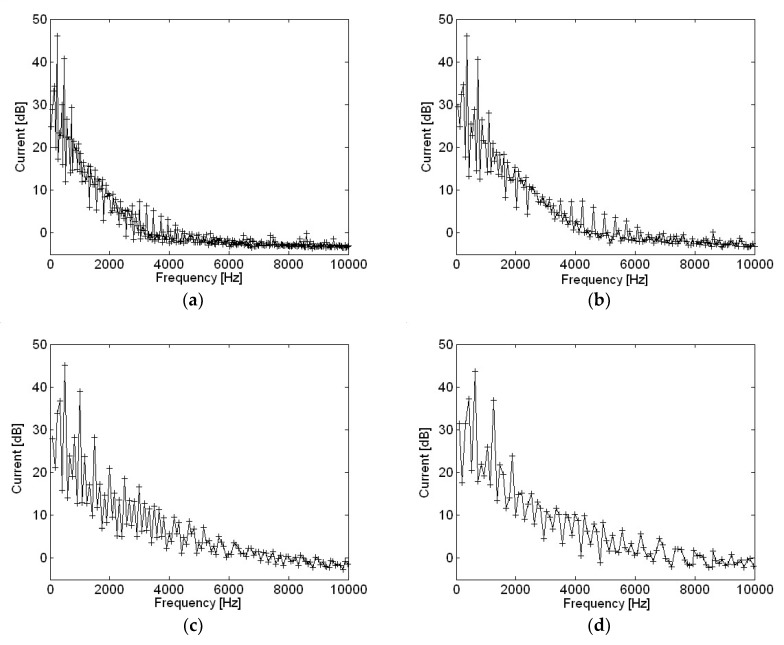
Motor current measurement with different motor rotational speeds: (**a**) 2480 rpm; (**b**) 3720 rpm; (**c**) 4960 rpm; (**d**) 6200 rpm.

**Figure 5:**
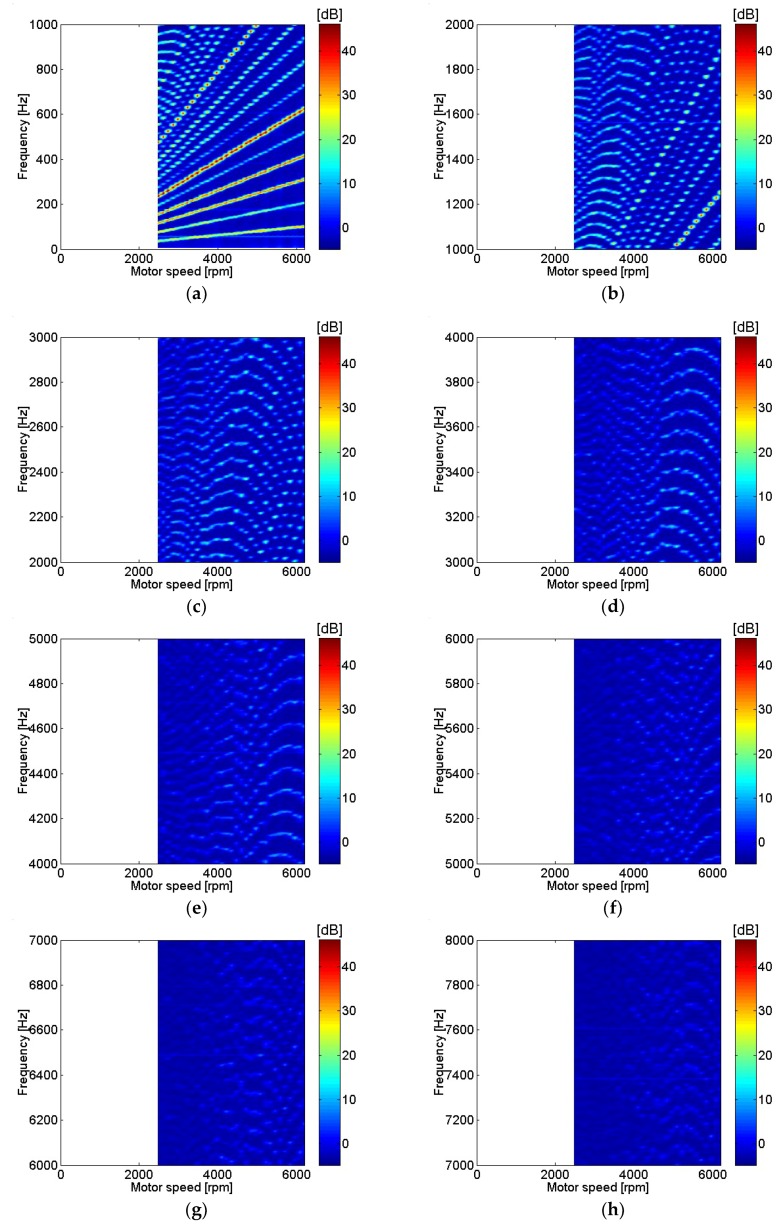
Motor current at various motor speeds: (**a**) 0–1 kHz; (**b**) 1–2 kHz; (**c**) 2–3 kHz; (**d**) 3–4 kHz; (**e**) 4–5 kHz; (**f**) 5–6 kHz; (**g**) 6–7 kHz; (**h**) 7–8 kHz.

Motor current measurements with different motor rotational speed are shown in Figure 4. Only frequencies corresponding to a multiple of rotational speed or fundamental frequencies are included since they represent points of interest with relatively higher input power. At low frequencies, the current level at major peaks was almost identical regardless of motor speed. In contrast, as motor speed rose, current level increase at high frequencies was dominant. Motor current was represented in dB using a reference value of 20 μA. Distortion of the current waveform at higher frequencies was clearly shown at higher motor speeds. Motor current with various motor rotational speeds is shown in Figure 5. The range of motor speeds was between 2480 and 6200 rpm, which corresponded to a power supply voltage of 6 V and 15 V. Very clear motor current level was shown below 600 Hz, or sixth order of motor rotational speed. Motor current level decreased rapidly as frequency increased, and current level was significantly lower above 4 kHz.

**Figure 6:**
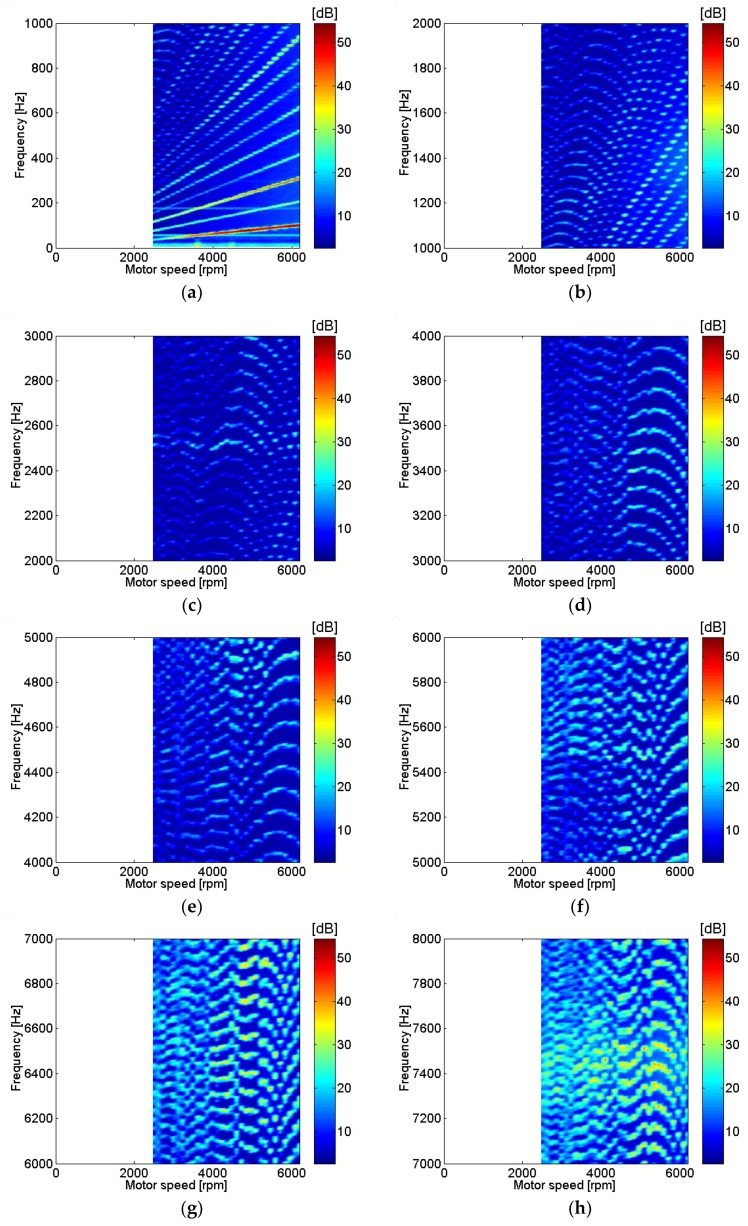
Sound pressure at various motor speeds: (**a**) 0–1 kHz; (**b**) 1–2 kHz; (**c**) 2–3 kHz; (**d**) 3–4 kHz; (**e**) 4–5 kHz; (**f**) 5–6 kHz; (**g**) 6–7 kHz; (**h**) 7–8 kHz.

**Figure 7:**
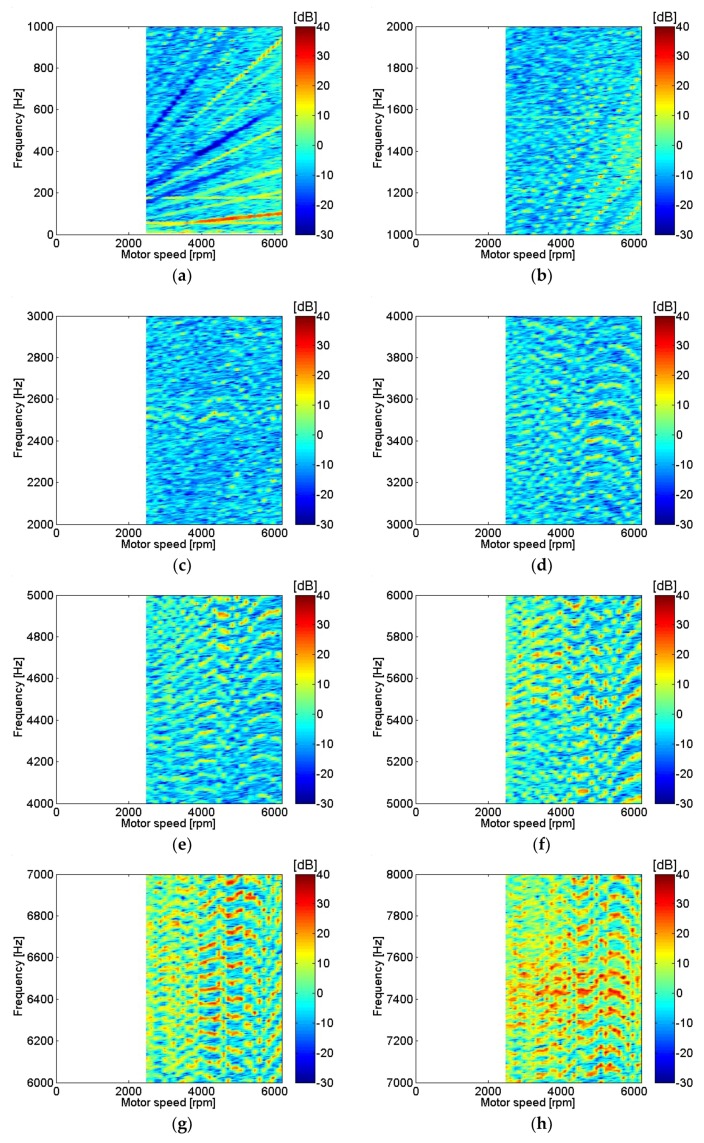
Transfer functions between sound pressure and motor current at various motor speeds: (**a**) 0–1 kHz; (**b**) 1–2 kHz; (**c**) 2–3 kHz; (**d**) 3–4 kHz; (**e**) 4–5 kHz; (**f**) 5–6 kHz; (**g**) 6–7 kHz; (**h**) 7–8 kHz.

Similarly, sound pressure at the center of the motor with various motor rotational speeds is shown in Figure 6. The sound pressure measurement location at the center of the motor corresponded to the position of microphone 3, as shown in Figure 1b. A relatively similar contour can be seen in current and sound pressure measurement results for frequencies below 1 kHz, as in Figure 5a and Figure 6a. In contrast, even though the current level was very low, the sound pressure measurement level was relatively high for frequencies above 7 kHz as shown in Figure 5h and Figure 6h. Transfer functions between current and sound pressure measurement at the motor center were estimated and are shown in Figure 7. One of the highest peaks for the transfer functions at the fundamental frequencies of motor rotational speed, possibly related to unbalanced forces in the motor, is clearly shown in Figure 7a. Also, the peaks for the transfer functions at frequencies above 7 kHz, possibly related to resonance of the motor housing, are clearly shown in Figure 7h [6].

**Figure 8:**
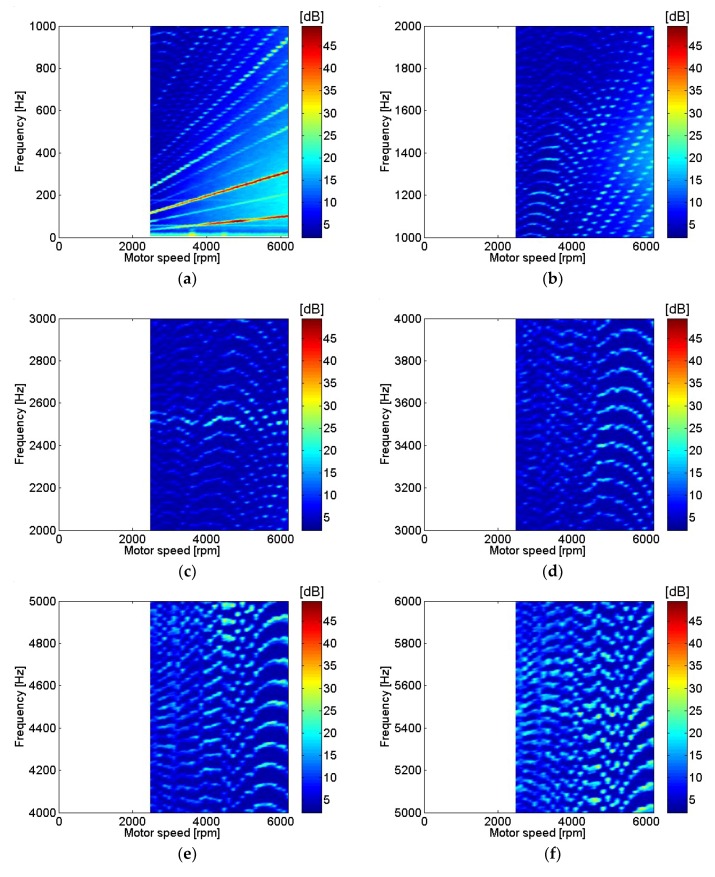

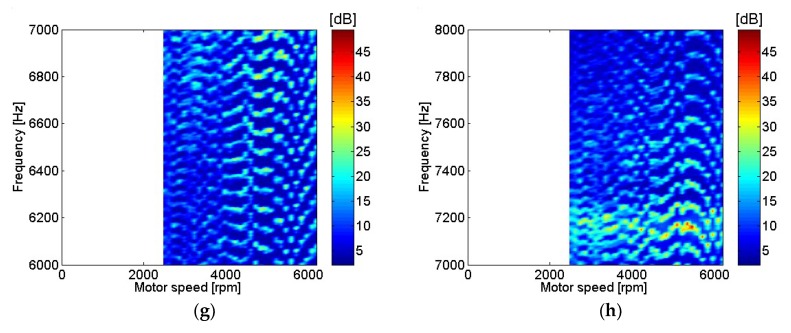
Sound pressure on top at various motor speeds: (**a**) 0–1 kHz; (**b**) 1–2 kHz; (**c**) 2–3 kHz; (**d**) 3–4 kHz; (**e**) 4–5 kHz; (**f**) 5–6 kHz; (**g**) 6–7 kHz; (**h**) 7–8 kHz.

**Figure 9:**
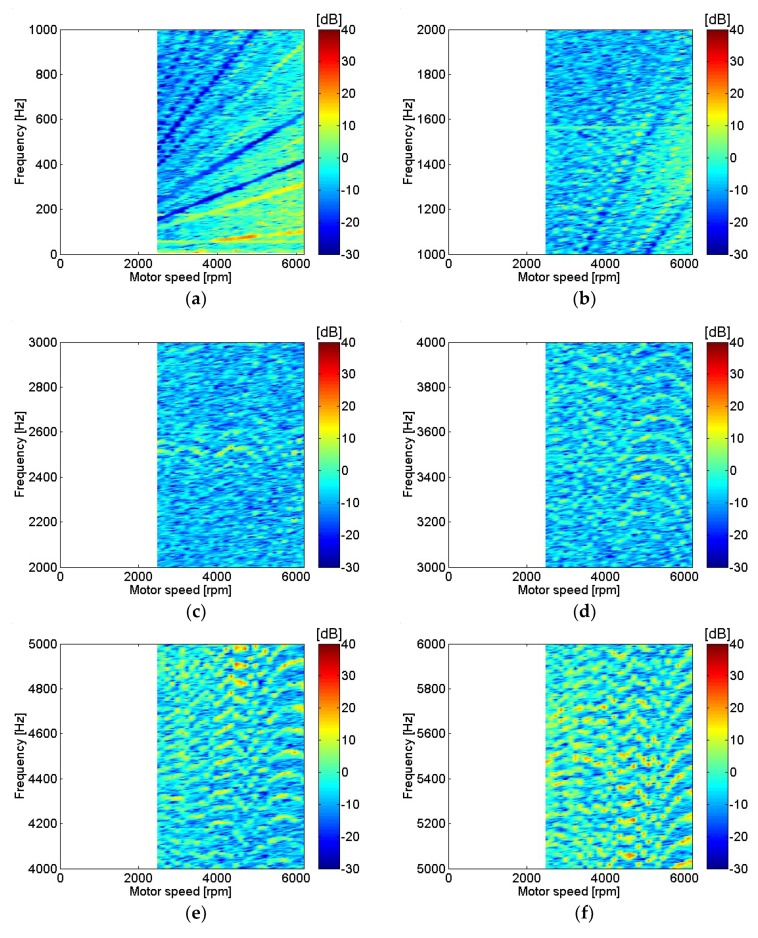

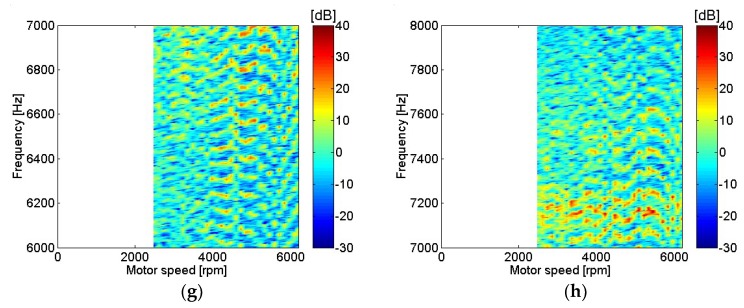
Transfer functions between sound pressure and motor current on top at various motor speeds: (**a**) 0–1 kHz; (**b**) 1–2 kHz; (**c**) 2–3 kHz; (**d**) 3–4 kHz; (**e**) 4–5 kHz; (**f**) 5–6 kHz; (**g**) 6–7 kHz; (**h**) 7–8 kHz.

**Figure 10:**
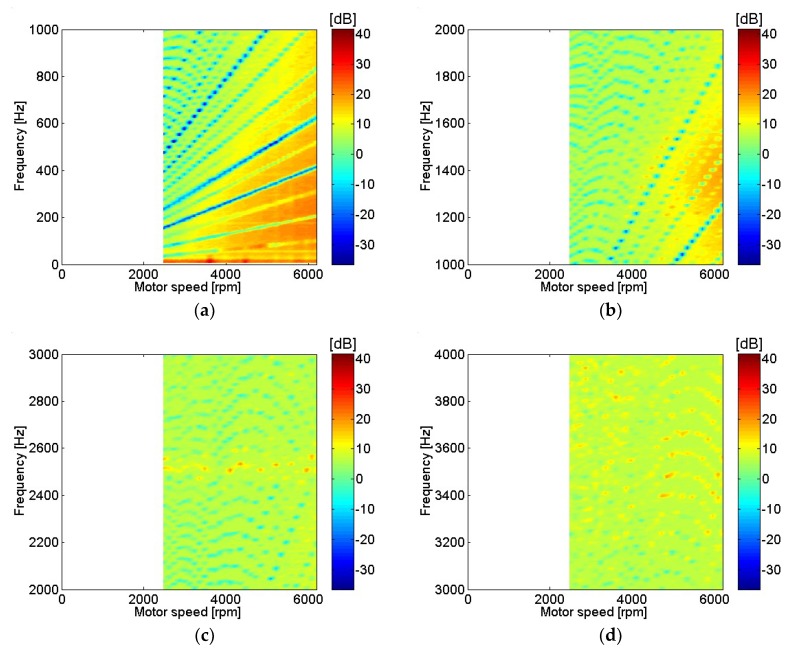

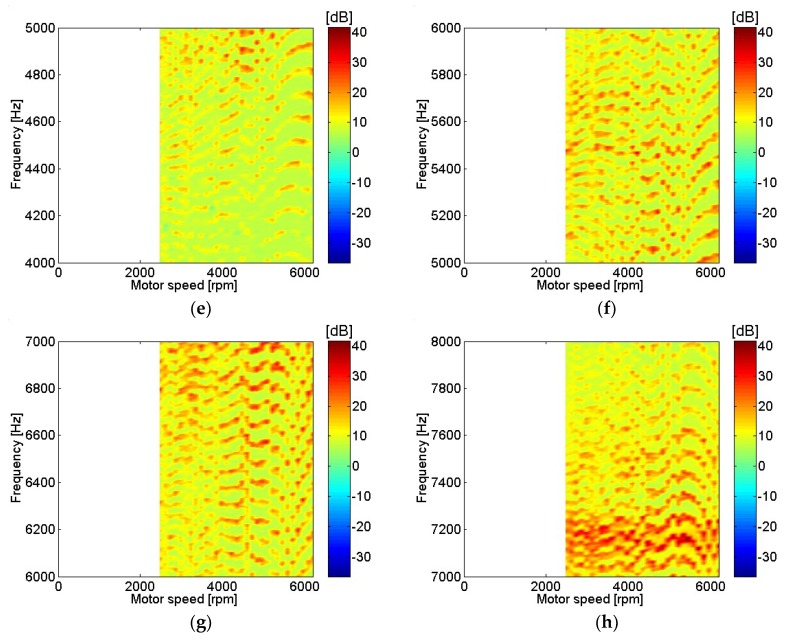
Amplitude ratio of sound pressure and motor current on top at various motor speeds: (**a**) 0–1 kHz; (**b**) 1–2 kHz; (**c**) 2–3 kHz; (**d**) 3–4 kHz; (**e**) 4–5 kHz; (**f**) 5–6 kHz; (**g**) 6–7 kHz; (**h**) 7–8 kHz.

Sound pressure at the top of the motor at various motor rotational speeds is shown in Figure 8. The sound pressure measurement location at the top of the motor corresponded to the position of microphone 4, as shown in Figure 1b. The relatively similar contour between results is shown in sound pressure measurements from different locations around the motor center and top in the frequency range below 1 kHz, as shown in Figure 6a and Figure 8a. The sound pressure measurement level was relatively high at frequencies around 7.2 kHz, as shown in Figure 8h. The transfer functions between current and sound pressure measurement at the top of the motor were estimated and are shown in Figure 9. The magnitude of the transfer function level was highest at frequencies around 7.2 kHz, as shown in Figure 9h. The amplitude ratio between sound pressure and current measurement at the top of the motor at various motor speeds is shown in Figure 10.

**Figure 11:**
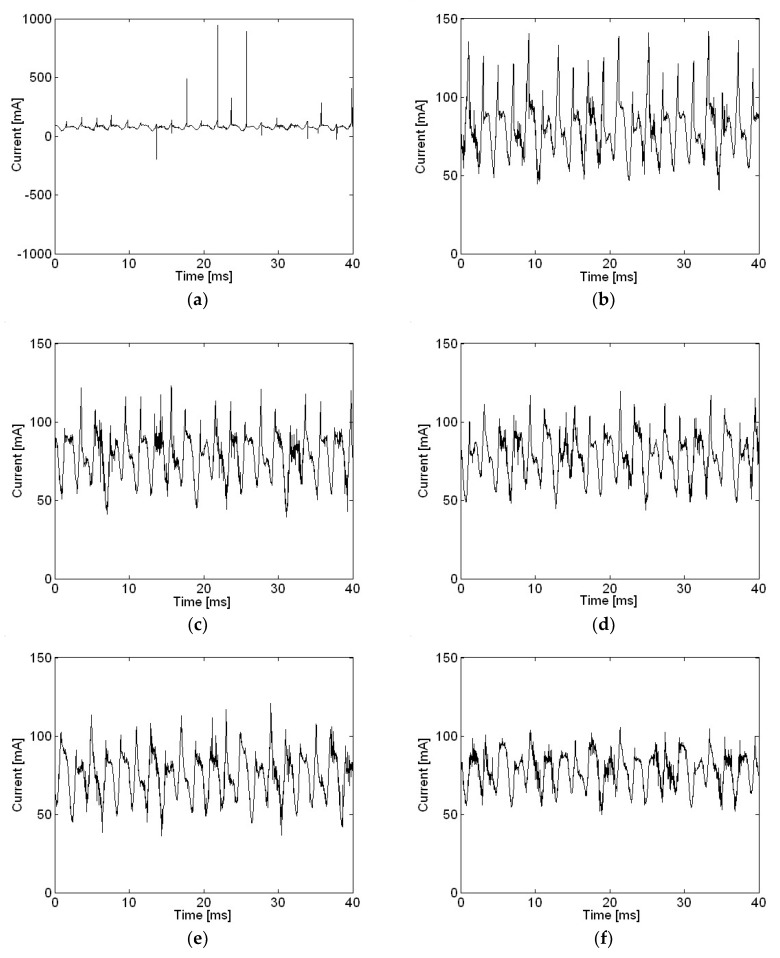
Motor current with various capacitances in the time domain: (**a**) 0; (**b**) 10 μF; (**c**) 33 μF; (**d**) 47 μF; (**e**) 100 μF; (**f**) 220 μF.

Motor current measurements with various capacitances in the time domain are shown in Figure 11. The resistance value shown in Figure 1 and Figure 2 remained constant at 0.1 Ω for all measurements in this study. However, the value of capacitance was increased from 0 to 220 μF to investigate changes in the motor current waveform and radiated sound pressure. Except for a capacitance of 0, which represented the circuit without any capacitance, other entries represent the actual values of capacitance according to the capacitor. The actual value of capacitance connected to the motor power input was 100 μF for all cases, if not described otherwise in present study. Very high spikes in current were shown when no capacitor was included, as in Figure 11a. One reason behind the decision to include a capacitor for motor input power was to remove such spikes in motor current measurements, to prevent aliasing when sampling at a rate of 44.1 kHz was very low compared to the sharpness of spikes. However, a sampling rate of 44.1 kHz is reasonably high enough for sound pressure measurement. Another reason was to reduce radiated sound pressure by removing spikes in the motor input current. Even by including the smallest capacitance, such as 10 μF, spikes in current measurement were significantly reduced, as shown in Figure 11b. Spikes in current measurement were further reduced as capacitance was increased, and the lowest spikes are shown in Figure 11f when a capacitance of 220 μF, the greatest in this research, was used.

A relatively higher amplitude ratio between sound pressure and current measurement appeared below 1 kHz, around 1.4 kHz, above 5 kHz. The amplitude ratio was highest around 7.2 kHz and represented modal resonances, as shown in Figure 10.

**Figure 12:**
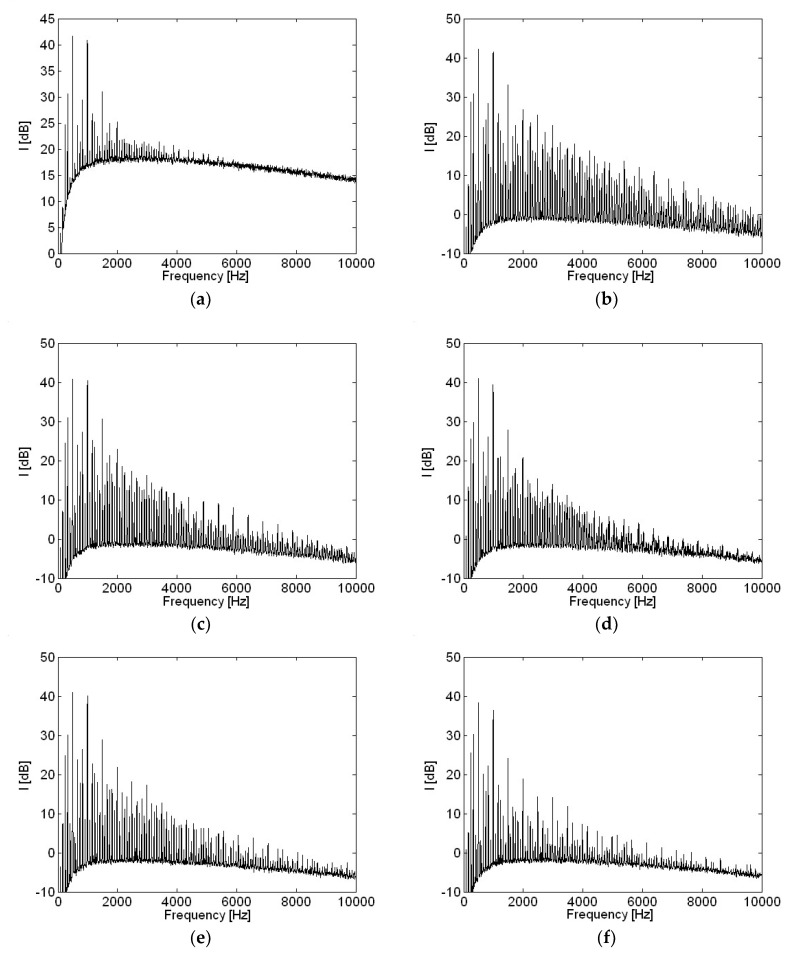
Motor current measurement with various capacitances in the frequency domain: (**a**) 0; (**b**) 10 μF; (**c**) 33 μF; (**d**) 47 μF; (**e**) 100 μF; (**f**) 220 μF.

Motor current measurements with various capacitances in the frequency domain are shown in Figure 12. Current level at higher frequencies reduced as capacitance becomes higher, when compared with a current measurement at 10 μF capacitance. However, little change occurred at two major frequencies with the two highest amplitudes, such as 500 Hz and 1000 Hz shown in Figure 12, as capacitance increased, except for at 220 μF. There was slightly more change in current level at these two major frequencies for capacitance of 220 μF than for other, smaller capacitances.

**Figure 13:**
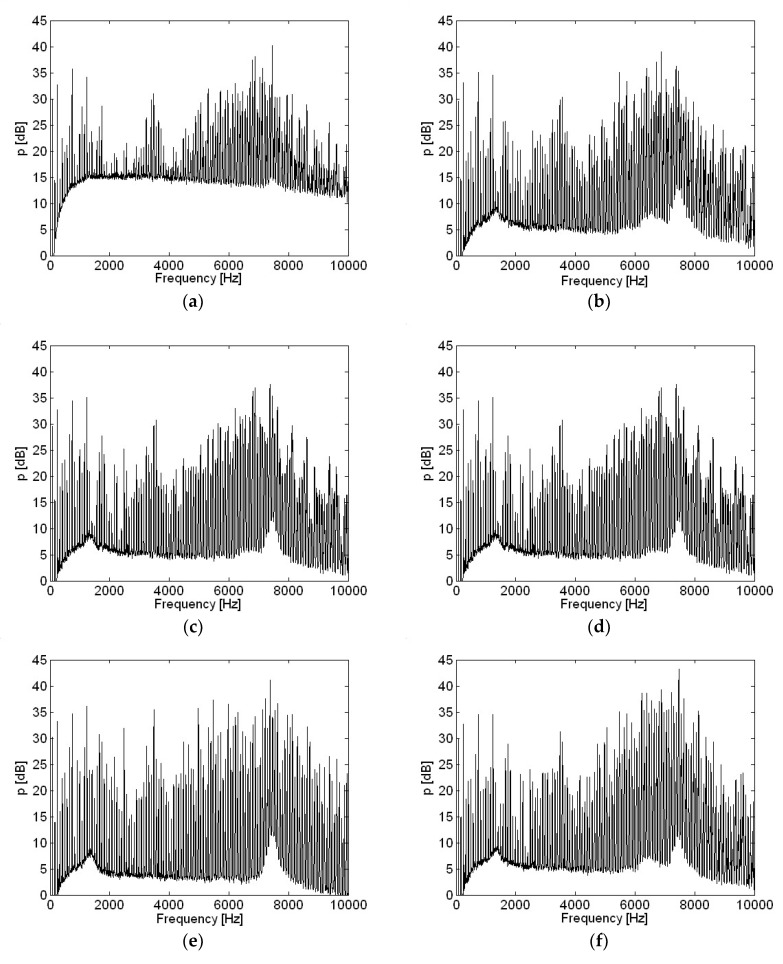
Amplitude of sound pressure at the motor center with various capacitances: (**a**) 0; (**b**) 10 μF; (**c**) 33 μF; (**d**) 47 μF; (**e**) 100 μF; (**f**) 220 μF.

**Figure 14:**
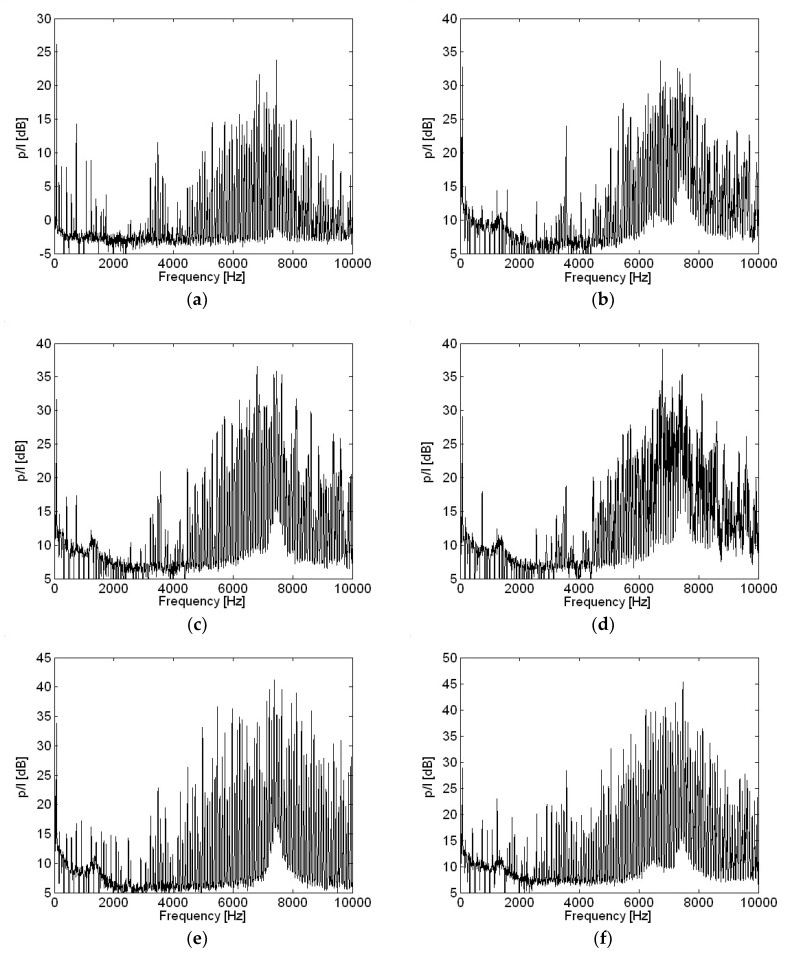
Amplitude ratio of sound pressure at the motor center and motor current with various capacitances: (**a**) 0; (**b**) 10 μF; (**c**) 33 μF; (**d**) 47 μF; (**e**) 100 μF; (**f**) 220 μF.

The amplitude of the sound pressure measurement at the motor center with various capacitances is shown in Figure 13. However, significant changes in sound pressure level at the motor center with various capacitances was not clearly shown. The amplitude ratio for sound pressure at the motor center and motor current with various capacitances is shown in Figure 14. There was significant deviation in the amplitude ratio with various capacitances, but the highest peaks occurred at frequencies around 7 kHz for all capacitances, as shown in Figure 14.

**Figure 15:**
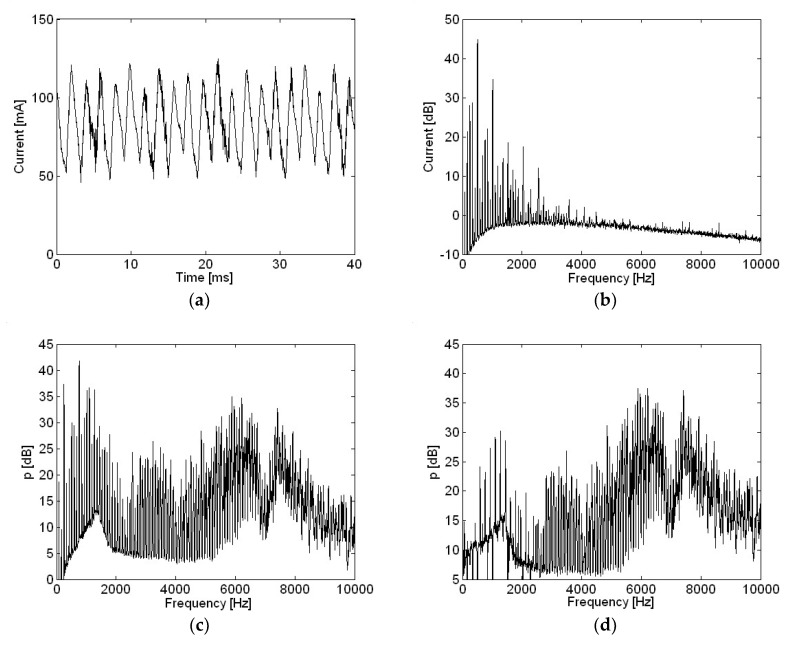
Motor rotating clock-wise with a 100 μF capacitor: (**a**) current in the time domain; (**b**) current in the frequency domain; (**c**) sound pressure; (**d**) amplitude ratio of sound pressure and current.

Motor current, sound pressure, and the amplitude ratio for sound pressure and current for a motor rotating clock-wise (CW) with a 100 μF capacitor are shown in Figure 15. Significant differences in motor current for opposite rotational directions were shown both in the time and frequency domains in Figure 11e and Figure 15a and Figure 12e and Figure 15b, respectively. One possible explanation for this difference in motor current given opposite rotational directions would be a deviation in the condition of brushes and commutators. Current and sound pressure levels for a motor with various capacitances, summed over the frequency range from 0 to 10 kHz, are shown in Table 1. The four different measurement locations from P1 to P4 in Table 1 coincide with microphone locations from 1 to 4 as shown in Figure 1. The mean refers to the root-mean-square of sound pressure measurements in four positions for each capacitance. Here, the accuracy of the instrumentation should also be considered, which is typically more or less than one dB. So, the decimal points in dB do not represent accuracy of the results. The ranking of sound pressure in terms of capacitance depended on the measurement location. Overall, the mean sound pressure with a capacitance of 47 μF was slightly lower than that at 100 μF; 57.1 dB and 57.3 dB, respectively. Mean sound pressure of 1.1 dB and 0.9 dB was reduced with capacitances of 47 μF and 100 μF when compared with sound pressure without a capacitor. However, even though the current level was further reduced by using a higher capacitance of 220 μF, mean sound pressure was not reduced when compared with sound pressure without a capacitor. Also, even though there were current level differences between CCW and CW motor rotational directions, mean sound pressure did not change due to motor rotational directions.

**Figure 16:**
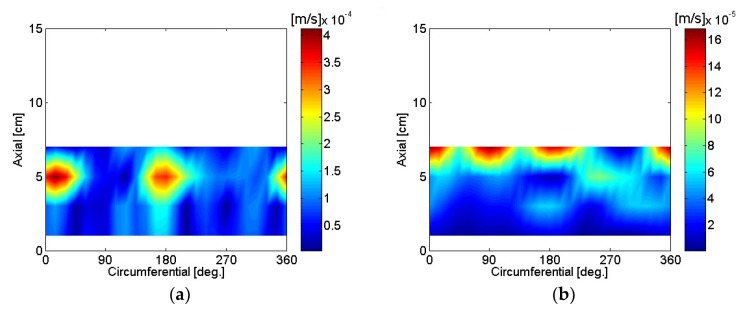

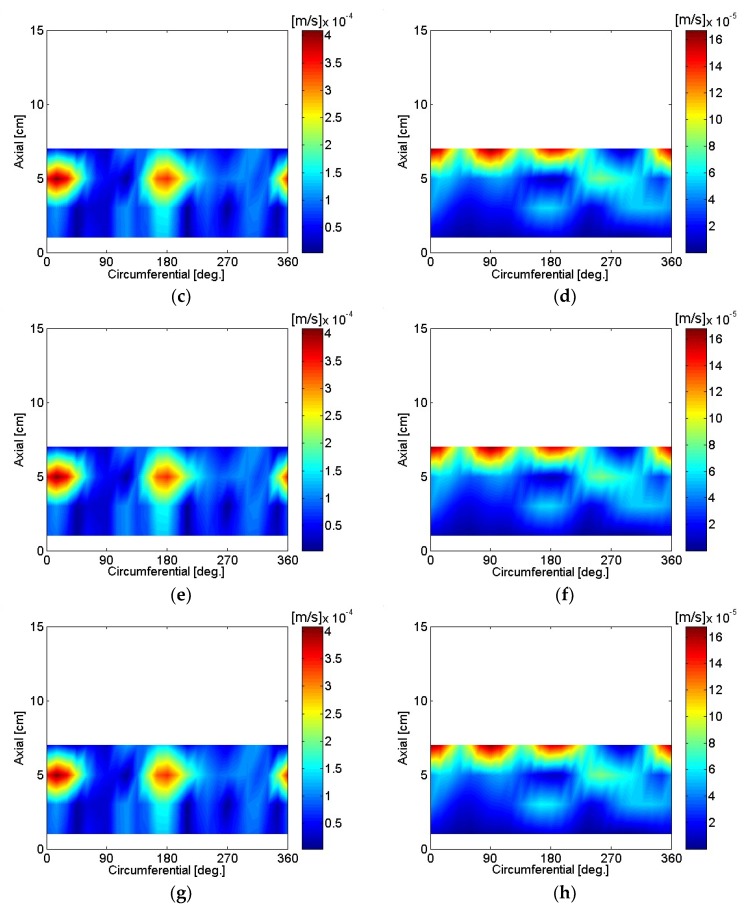
Particle velocity of the motor: (**a**) 80 Hz, current reference; (**b**) 244 Hz, current reference; (**c**) 80 Hz, moving reference 3; (**d**) 244 Hz, moving reference 3; (**e**) 80 Hz, moving reference 4; (**f**) 244 Hz, moving reference 4; (**g**) 80 Hz, auto moving reference; (**h**) 244 Hz, auto moving reference.

**Figure 17:**
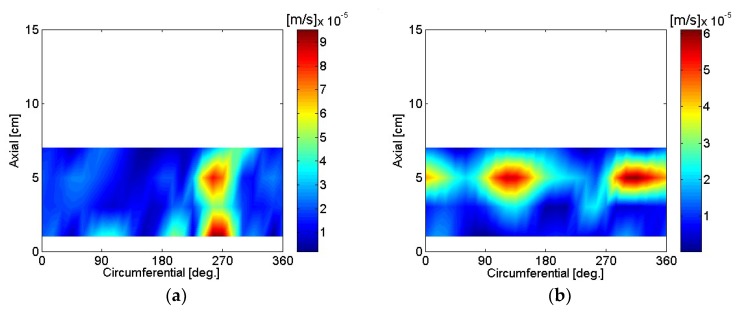

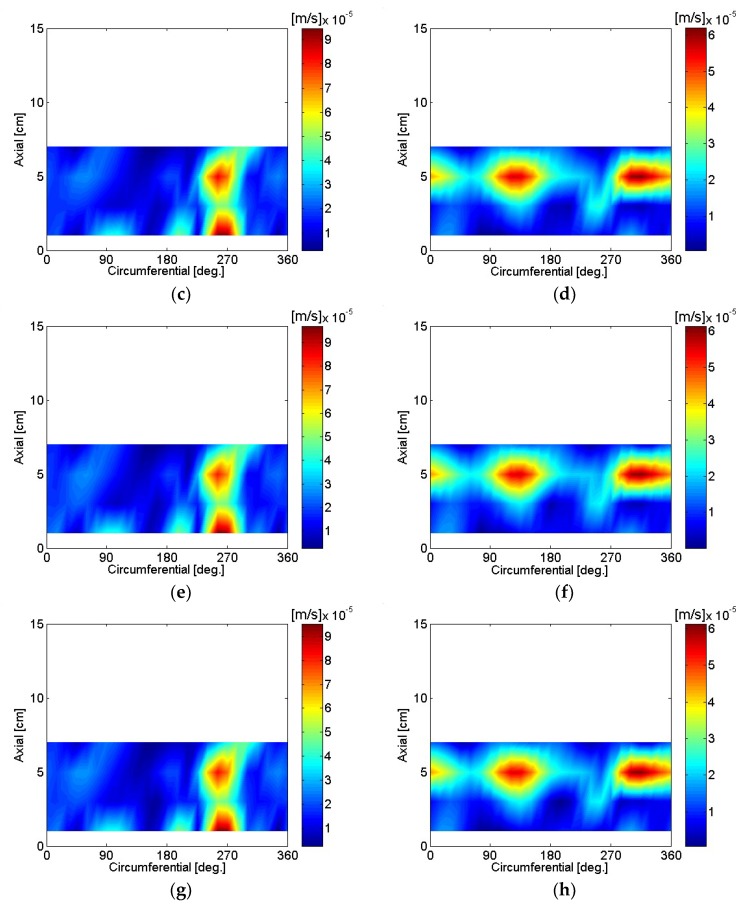
Particle velocity of the motor: (**a**) 496 Hz, current reference; (**b**) 744 Hz, current reference; (**c**) 496 Hz, moving reference 3; (**d**) 744 Hz, moving reference 3; (**e**) 496 Hz, moving reference 4; (**f**) 744 Hz, moving reference 4; (**g**) 496 Hz, auto moving reference; (**h**) 744 Hz, auto moving reference.

**Figure 18:**
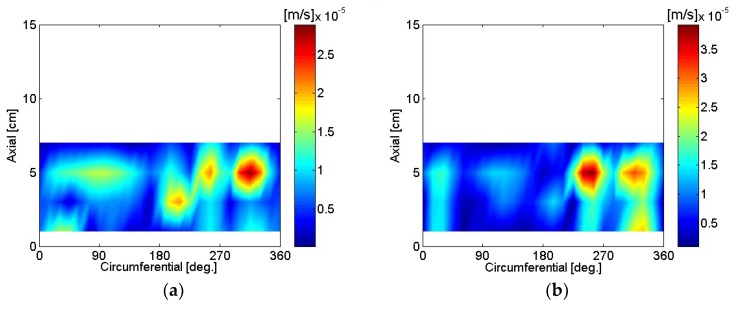

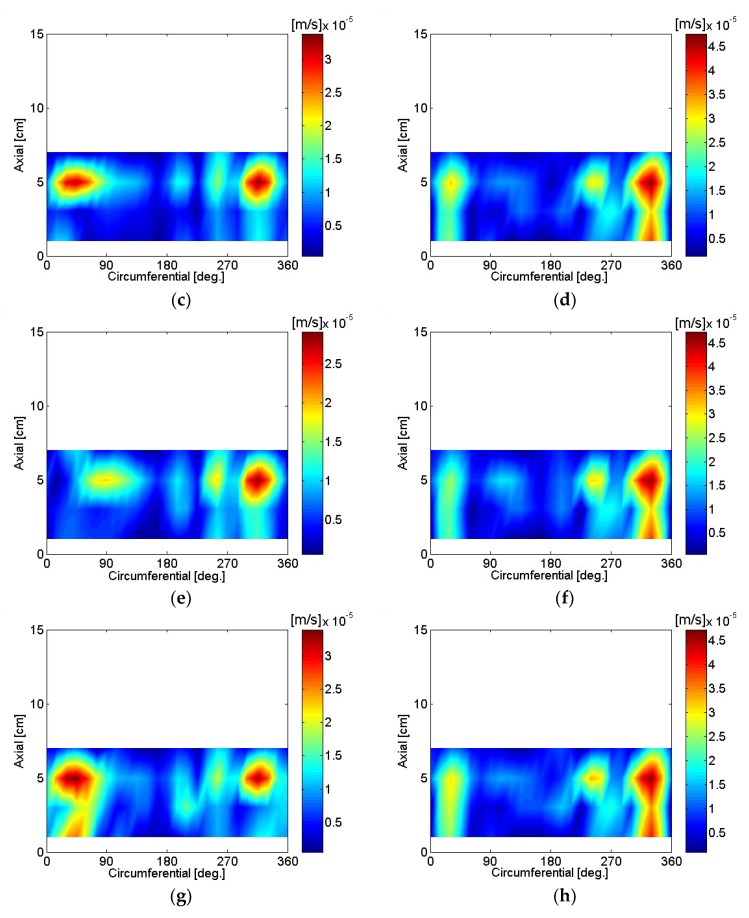
Particle velocity of the motor: (**a**) 996 Hz, current reference; (**b**) 1244 Hz, current reference; (**c**) 996 Hz, moving reference 3; (**d**) 1244 Hz, moving reference 3; (**e**) 996 Hz, moving reference 4; (**f**) 1244 Hz, moving reference 4; (**g**) 996 Hz, auto moving reference; (**h**) 1244 Hz, auto moving reference.

**Figure 19:**
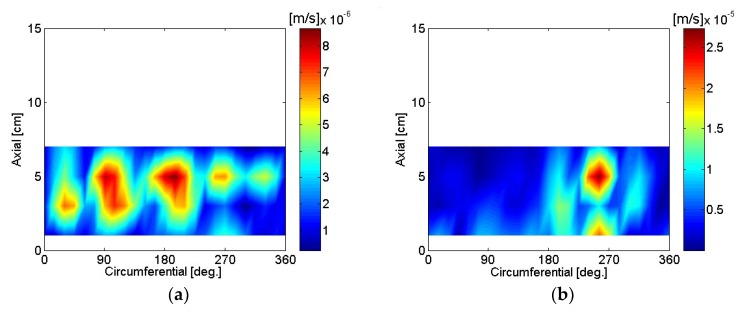

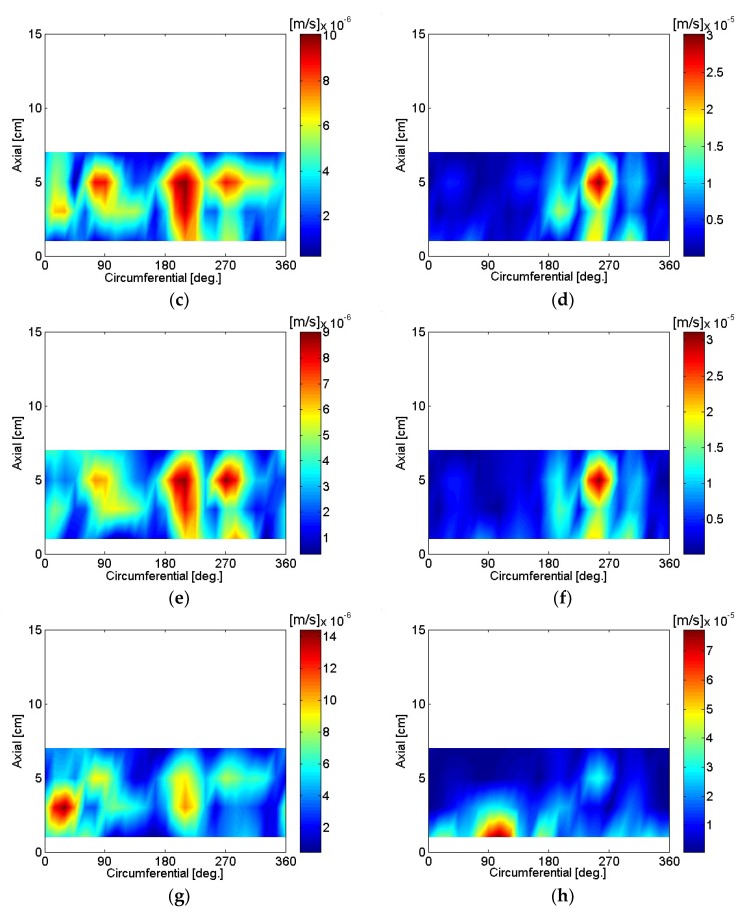
Particle velocity of the motor: (**a**) 1328 Hz, current reference; (**b**) 1736 Hz, current reference; (**c**) 1328 Hz, moving reference 3; (**d**) 1736 Hz, moving reference 3; (**e**) 1328 Hz, moving reference 4; (**f**) 1736 Hz, moving reference 4; (**g**) 1328 Hz, auto moving reference; (**h**) 1736 Hz, auto moving reference.

**Figure 20:**
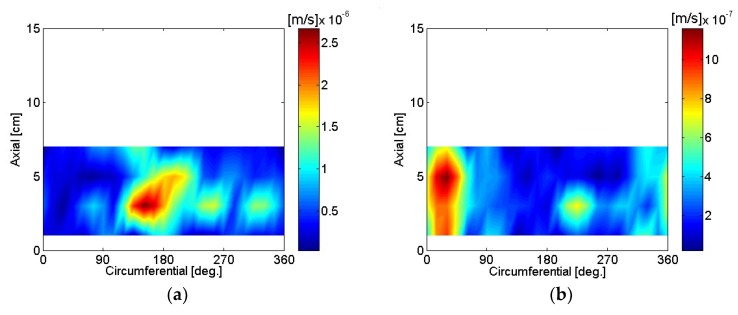

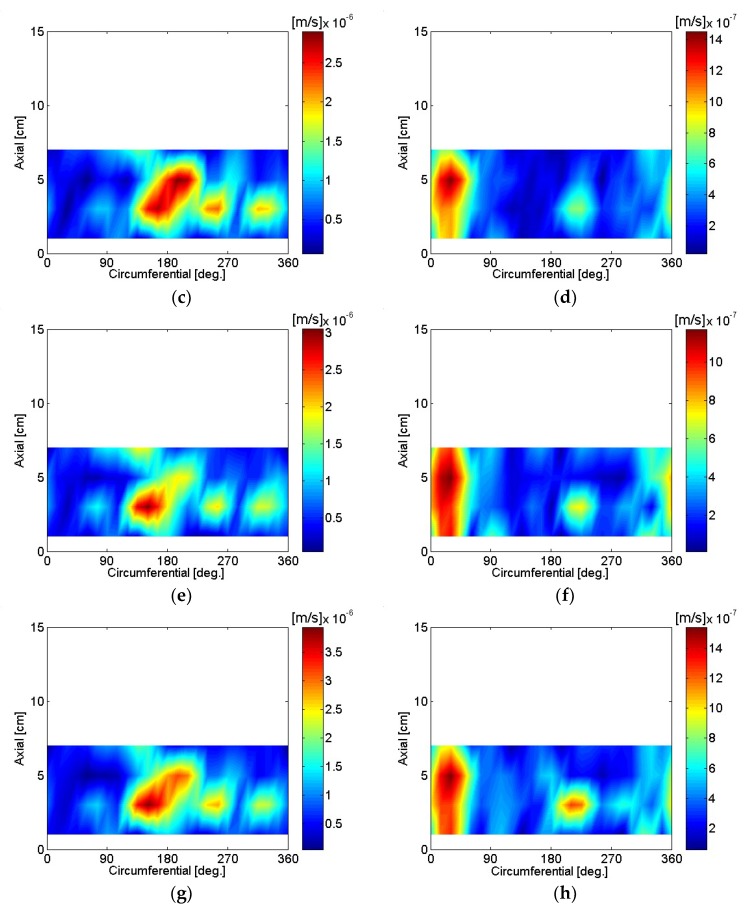
Particle velocity of the motor: (**a**) 4988 Hz, current reference; (**b**) 5048 Hz, current reference; (**c**) 4988 Hz, moving reference 3; (**d**) 5048 Hz, moving reference 3; (**e**) 4988 Hz, moving reference 4; (**f**) 5048 Hz, moving reference 4; (**g**) 4988 Hz, auto moving reference; (**h**) 5048 Hz, auto moving reference.

**Figure 21:**
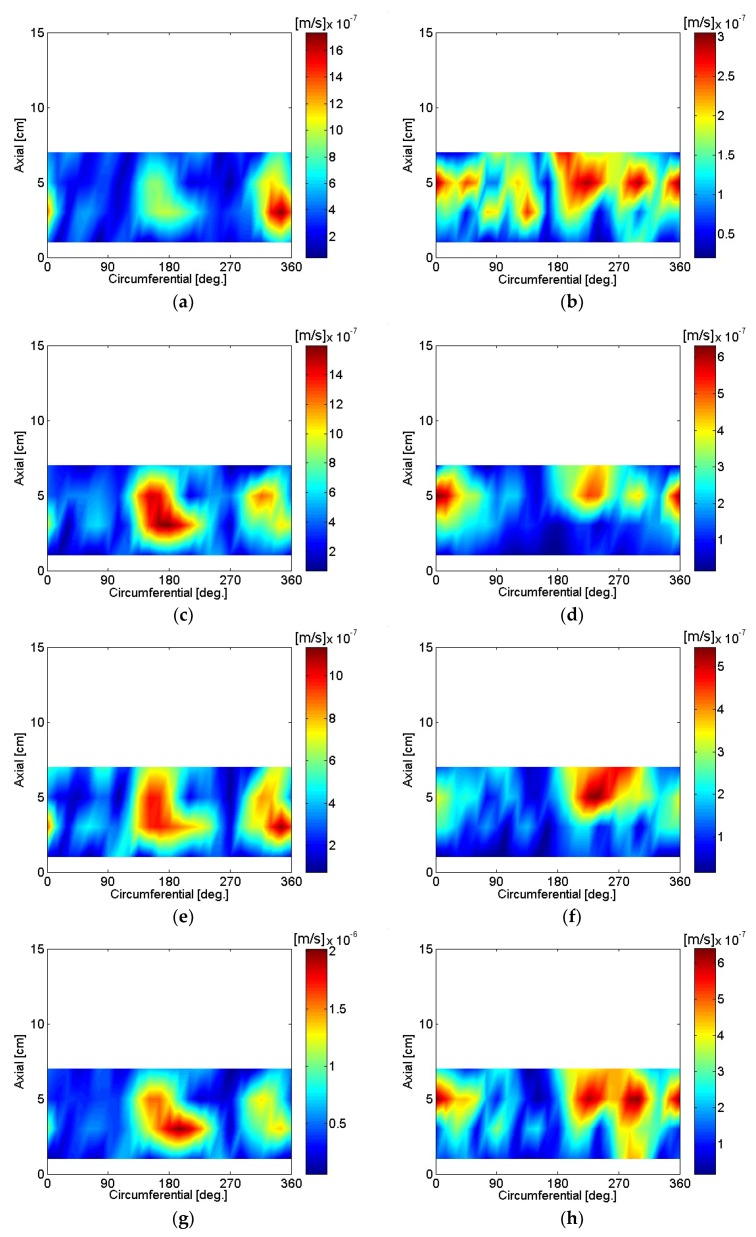
Particle velocity of the motor: (**a**) 5072 Hz, current reference; (**b**) 7208 Hz, current reference; (**c**) 5072 Hz, moving reference 3; (**d**) 7208 Hz, moving reference 3; (**e**) 5072 Hz, moving reference 4; (**f**) 7208 Hz, moving reference 4; (**g**) 5072 Hz, auto moving reference; (**h**) 7208 Hz, auto moving reference.

The particle velocity of the motor housing surface was reconstructed using SONAH and is shown in Figure 16, Figure 17, Figure 18, Figure 19, Figure 20 and Figure 21. All reconstructed particle velocity data in this study was based on A-weighted measurement pressure. Results from four different types of references (current measurement, a moving reference at the motor center, a moving reference on top of the motor, and an auto moving reference) are shown for twelve frequencies. Results from the moving references were estimated using Equation (14) by combining current and sound pressure measurements using scanning microphones at moving reference locations. The moving reference locations coincided with scanning microphone positions as shown in Figure 1. The auto moving reference was a special case described in this research, and each measurement from a scanning microphone was considered as a moving reference. Reconstructed particle velocity based on different types of references was relatively similar for most frequencies and agreed very well for low frequencies. However, deviations between reconstructed particle velocities using different types of references are shown at 996 Hz in Figure 18. It was challenging to judge which reference type was best at 996 Hz alone, even though some response of the motor housing due to electro-magnetic forces was shown for all four reference types. Also, deviations between reconstructed particle velocities using different types of references are shown at 1328 Hz in Figure 19. Reconstructed particle velocity using moving reference 3, located at the motor center, provides a visualization of motor internal resonance that is slightly more clear than from other reference types. Reconstructed particle velocities from different types of references were similar except for the results of the auto moving reference, which was dominated by a reflection from the base plate at 1736 Hz. These similar reconstructed particle velocities provided clear visualizations of *n* = 2 mode at the bottom of the motor housing at 4988 Hz [6]. Which reference type was best at 5072 Hz alone was not obvious, even though some response of the motor housing due to electro-magnetic forces at the bottom of the motor housing was shown for all four reference types. Reconstructed particle velocity using moving reference 3, located at the motor center, provided a visualization of *n* = 2 mode at the center of the motor housing that was slightly more clear than that of other reference types at 7208 Hz. A summary of source particle velocity reconstruction is provided in Table 2. Reconstructed particle velocities based on different types of references were relatively similar for the twelve frequencies shown, with some exceptions, especially at the highest frequency and frequencies corresponding to internal resonance of the motor.

**Figure 22:**
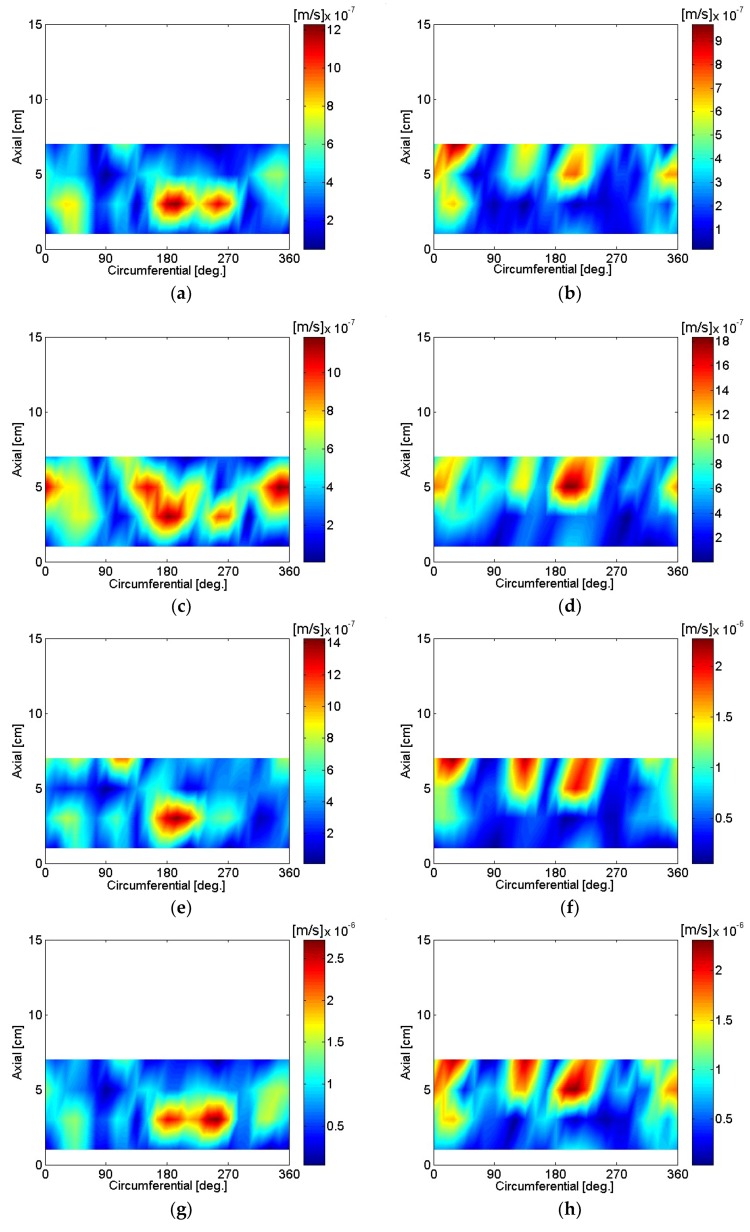
Particle velocity of the motor, CW: (**a**) 5120 Hz, current reference; (**b**) 7172 Hz, current reference; (**c**) 5120 Hz, moving reference 3; (**d**) 7172 Hz, moving reference 3; (**e**) 5120 Hz, moving reference 4; (**f**) 7172 Hz, moving reference 4; (**g**) 5120 Hz, auto moving reference; (**h**) 7172 Hz, auto moving reference.

Sound pressure measurements were taken with the motor rotating CW. The motor was powered to rotate in the opposite direction by switching the poles of the motor power input. The particle velocity of the motor housing surface while rotating CW was reconstructed using SONAH, and is shown in Figure 22. There was a slight deviation in motor rotational speed, 4960 rpm and 5080 rpm for CCW and CW, when the motor was rotating in different directions, with the power supply output voltage kept the same at 12 V. Reconstructed particle velocity using moving reference 3, located at the motor center, provided a visualization of the motor’s internal resonance that was slightly clearer than that of other reference types, based on reconstructed particle velocity of the motor housing surface at 5120 Hz and 7172 Hz.

The mean sound pressure measurement level for both in space and at frequencies above 0 to 10 kHz was estimated for motor rotational directions of CCW and CW. The mean of A-weighted measurement pressure was 58.9 dB and 58.6 dB, for CCW and CW. However, the mean of measurement pressure without weighting (with linear weighting) was 72.1 dB for both CCW and CW.

## 6. Conclusions

Small motor noise was measured without a reference microphone, and noise and vibration sources for a small DC motor were clearly visualized. As motors become increasingly smaller, the optimal position for a stationary reference microphone may not be accessible due to potential physical interference with scanning microphones. Current measurement for the motor was used as a reference signal, and strategies for small motor noise visualization without using a reference microphone were developed. Scanning microphone measurement signals were combined with current measurements and used as moving reference signals. Motor noise visualization results using current measurement as a reference signal and different moving reference locations were estimated and shown in this study. Also, sound pressure for a motor rotating both CCW and CW was measured over the entire surface to be visualized. Sound pressure and motor current were measured with various capacitance for motor power input. Both sound pressure and motor current were measured for a motor rotating at various speeds, and the transfer functions and ratio of amplitude between sound pressure and motor current were estimated.

Previously, sources of small DC motor noise were accurately visualized over a wide range of frequencies using acoustical holography, with a reference microphone located on top of the motor [6]. Small motor noise was measured and visualized using only current as a reference signal in this research, and it has been shown that it is ideal to use a microphone measurement as a reference rather than current measurement alone. However, noise sources (e.g., 1328 Hz and 7208 Hz) were visualized more clearly by implementing moving references than by using only current measurement as a reference, combining current and scanning microphone measurements close to the motor housing. 

Arcs in current measurement for the motor were significantly reduced by connecting a capacitor to the power input of the motor, and motor noise level was reduced by about one dB with an optimal capacitor. Even though significant differences existed in the frequency domain for a motor rotating CCW as opposed to CW, there was no difference in mean sound pressure level, especially with linear weighting of measurement pressure.

A run-up test of the motor with both sound pressure and current measurement captured motor resonances more clearly than sound pressure measurement alone. The transfer function and amplitude ratio of sound pressure and current reflected the motor internal and housing resonance clearly as well.

The high-frequency component of motor current reduced more rapidly than that of sound pressure, so using motor current measurement as a reference for noise source visualization and estimation was not ideal. When combined with sound pressure measurement, however, current measurement offered a reasonable reference in place of a reference microphone. Such a method for sound visualization would be appropriate for improving the source characterization of very small motors and structures where a reference microphone cannot be placed in an optimal location close to sources.

## Figures and Tables

**Table 1:** Current and sound level (dB) of a motor with various capacitances.

Capacitance	Direction	I	P1	P2	P3	P4	Mean
0	CCW	52.5	61.8	58.3	55.1	51.3	58.2
10 μF	CCW	48.7	61.5	58.3	54.0	48.7	57.8
33 μF	CCW	47.2	61.5	58.2	53.8	48.7	57.8
47 μF	CCW	46.5	61.4	56.2	52.9	48.1	57.1
100 μF	CCW	46.8	61.6	55.2	54.1	50.9	57.3
220 μF	CCW	44.1	61.6	58.6	55.5	49.9	58.2
100 μF	CW	48.3	61.5	55.0	54.4	51.1	57.3

**Table 2:** Description of source particle velocity reconstruction.

Frequency	Order	Description of Source
80 Hz	1st *f*_r_	Unbalanced force
244 Hz	3rd *f*_r_, 1st *f*_e_	Top ventilation
496 Hz	6th *f*_r_, 2nd *f*_e_	Electro-magnetic force
744 Hz	9th *f*_r_, 3rd *f*_e_	Electro-magnetic force
996 Hz	12th *f*_r_, 4th *f*_e_	Electro-magnetic force
1244 Hz	15th *f*_r_, 5th *f*_e_	Electro-magnetic force
1328 Hz	16th *f*_r_	Internal resonance
1736 Hz	21st *f*_r_, 7th *f*_e_	Electro-magnetic force
4988 Hz	60th *f*_r_, 20th *f*_e_	Housing bottom *n* = 2 mode
5072 Hz	61th *f*_r_	Internal resonance
7208 Hz	87th *f*_r_, 29th *f*_e_	Housing center *n* = 2 mode
